# Immunohistochemical detection of piscine reovirus (PRV) in hearts of Atlantic salmon coincide with the course of heart and skeletal muscle inflammation (HSMI)

**DOI:** 10.1186/1297-9716-43-27

**Published:** 2012-04-09

**Authors:** Øystein Wessel Finstad, Knut Falk, Marie Løvoll, Øystein Evensen, Espen Rimstad

**Affiliations:** 1 Department of Food Safety & Infection Biology, Norwegian School of Veterinary Science, Postboks 8146 Dep, N-0033 OSLO Norwegian, Norway; 2 Norwegian Veterinary Institute, Pb 750 Sentrum, N-0106 Oslo Norwegian, Norway; 3 Department of Basic Sciences and Aquatic Medicine, Norwegian School of Veterinary Science, Postboks 8146 Dep, N-0033 OSLO Norwegian, Norway

## Abstract

Aquaculture is the fastest growing food production sector in the world. However, the increased production has been accompanied by the emergence of infectious diseases. Heart and skeletal muscle inflammation (HSMI) is one example of an emerging disease in farmed Atlantic salmon (*Salmo salar L*). Since the first recognition as a disease entity in 1999 it has become a widespread and economically important disease in Norway. The disease was recently found to be associated with infection with a novel reovirus, piscine reovirus (PRV). The load of PRV, examined by RT-qPCR, correlated with severity of HSMI in naturally and experimentally infected salmon. The disease is characterized by epi-, endo- and myocarditis, myocardial necrosis, myositis and necrosis of the red skeletal muscle. The aim of this study was to investigate the presence of PRV antigens in heart tissue of Atlantic salmon and monitor the virus distribution in the heart during the disease development. This included target cell specificity, viral load and tissue location during an HSMI outbreak. Rabbit polyclonal antisera were raised against putative PRV capsid proteins μ1C and σ1 and used in immunohistochemical analysis of archived salmon heart tissue from an experimental infection. The results are consistent with the histopathological changes of HSMI and showed a sequential staining pattern with PRV antigens initially present in leukocyte-like cells and subsequently in cardiomyocytes in the heart ventricle. Our results confirm the association between PRV and HSMI, and strengthen the hypothesis of PRV being the causative agent of HSMI. Immunohistochemical detection of PRV antigens will be beneficial for the understanding of the pathogenesis of HSMI as well as for diagnostic purposes.

## Introduction

Aquaculture is the fastest growing food producing sector in the world, and fish farming will be a key contributor to meet the growing demand for animal proteins [1]. However, intensive production, including rearing fish in dense populations, has led to the emergence of several new infectious diseases. Heart and skeletal muscle inflammation (HSMI), first detected in 1999, is an increasingly important disease in farmed Atlantic salmon (*Salmo salar L*.) [2]. HSMI usually occurs 5-9 months after transfer of the fish to seawater, and is characterized by epi-, endo- and myocarditis, myocardial necrosis, myositis and necrosis of the red skeletal muscle [2,3]. The cumulative mortality may reach 20%, but the morbidity is higher as most fish in an affected sea cage show histopathological lesions in the heart [4]. Pathological changes in the heart are also seen in other diseases in Atlantic salmon, including pancreas disease (PD) and cardiomyopathy syndrome (CMS) [5-8].

HSMI was recently found to be associated with a novel reovirus, piscine reovirus (PRV). The virus genome was identified using high throughput sequencing. Cultivation of PRV in commonly used fish cell lines has not been successful so far. The load of PRV, as measured by RT-qPCR, correlates with disease development in both naturally and experimentally infected salmon [9]. However, PRV is found to be ubiquitously distributed in healthy farmed Atlantic salmon, although at a much lower level than in diseased fish. PRV is also found in low quantities in wild Atlantic salmon [9].

The PRV belongs to the family *Reoviridae*, but it has not yet been classified at genus level. Phylogenetic analysis of derived amino acid sequences of the open reading frames of each genome segment indicated that PRV branches off the common root of the orthoreovirus and aquareovirus genera [9]. Like the orthoreoviruses, PRV contains 10 dsRNA genome segments, while aquareoviruses have 11 segments [10]. Based on sequence homologies the annotation of the PRV proteins has been set to follow that of mammalian orthoreoviruses (MRV), but structural and functional properties of the PRV proteins have not been determined. The PRV genome segments are classified according to size including three large (L), three medium (M) and four small (S) segments which encode λ-, μ- and σ-proteins, respectively.

Orthoreoviruses are ubiquitously distributed in various niches, as is PRV in farmed Atlantic salmon. Although human orthoreovirus infections are generally benign, orthoreovirus infections in other species are associated with a number of disease states [11]. Avian orthoreovirus infection in poultry is of pathogenic significance and is associated with myocarditis in chicken [12,13] and turkey poults [14,15]. Both poultry and aquaculture production confine animals under high density, conditions that facilitate transmission of infectious agents and induce stress and reduced resistance to disease. Reovirus induced myocarditis has also been studied in neonatal mice, where infection with MRV is a well characterized model of viral myocarditis [16-18].

In the present study we investigated the significance of PRV during development of the myocarditis in HSMI. *E.coli *recombinants of putative PRV outer capsid proteins σ1 and μ1C were produced for immunization of rabbits. The rabbit antisera were used for immunohistochemical detection of PRV in heart tissue of Atlantic salmon collected sequentially during an experimental HSMI infection. The development of the disease, including viral target cells, and load and tissue location of virus throughout the course of an HSMI outbreak were studied. Our results confirm the association between PRV and HSMI and strengthen the hypothesis of PRV being the causative agent of HSMI.

## Material and Methods

### Salmon samples

Formalin-fixed heart samples originated from a previously published experimental infection of HSMI [2]. Briefly, the experimental study included one group injected with tissue homogenate from HSMI diseased fish, one cohabitant group and one control group. Samples from five fish had been collected at each time point 2, 4, 6, 8, 10 and 12 weeks post infection (wpi).

### Protein expression and immunization

Total RNA was isolated from heart and kidney tissue from Atlantic salmon with HSMI using RNeasy Lipid Tissue Mini Kit (Qiagen GmBH, Hilden, Germany) according to the manufacturer's instructions. RT-PCRs were run (OneStep RT-PCR kit, Qiagen GmBH) to amplify the open reading frames (ORFs) of outer capsid proteins σ1 and μ1 of genome fragments S1 and M2, respectively. Primer sequences are listed in Table 1. The μ1C was amplified with a semi nested PCR, first the μ1 ORF and in the successive run the carboxy-terminal part, μ1C. An ATG overhang was introduced at the conserved cleavage site. The PCR products were cloned into pET100/D-TOPO (Invitrogen, Carlsbad, CA, USA) and the sequence was verified by Sanger sequencing (GATC Biotech AG, Konstanz, Germany). The pET100-σ1 and -μ1C plasmids were transfected into *E.coli *(BL21 DE3 strain, Invitrogen) and expressed as N-terminal (His)6 tagged fusion proteins, according to the manufacturer's instructions. The cultures were induced with isopropyl-d-thiogalacto-pyranoside (IPTG) and the protein expression was monitored by sodium dodecyl sulfate polyacrylamide gel electrophoresis (SDS-PAGE).

**Table 1:** Primers used in this study.

Primer names	Primer sequence (5' → 3')
σ1 S1-F	CACCATGCATAGATTTACCCAAGAAGACCA
σ1 S1-R	CTAGATGATGATCACGAAGTCTCCA
μ1 M2-F	CACCATGGGTAACTATCAGACAAGTAACAACCA
μ1 M2-R	GGATCCCTATTTTTGGCCTCGACGTGAGT
μ1C M2-F	CACC**ATG**CCTGGTGGTCACATGTATGTGATAT

Overhangs for directional cloning are underlined. The introduced start codon is shown in bold.

The recombinant proteins were purified using Ni-NTA purification system (Invitrogen). The cells were thawed on ice, resuspended in guanidinium lysis buffer [6 M Guanidine Hydrochloride, 20 mM Sodium Phosphate, 500 mM NaCl, pH 7.8], incubated for 30 min and sonicated on ice with three 5-sec pulses. The suspension was centrifuged at 3000 × *g *for 15 min at 4 °C. The following procedure was performed at room temperature for μ1C and at 4 °C for σ1: The supernatant was transferred to a Ni-NTA resin column equilibrated with denaturing binding buffer [8 M Urea, 20 mM Sodium Phosphate, pH 7.8]. The suspension was rotated for 30 min at room temperature and washed once with denaturing binding buffer, twice with denaturing wash buffer [8 M Urea, 20 mM Sodium Phosphate, 500 mM NaCl, pH 6.0] and four times with native wash buffer [50 mM sodium phosphate, 500 mM NaCl, 20 mM Imidazole, pH 8.0]. The μ1C and σ1 recombinant proteins were eluted with native elution buffer (pH 8.0) with 250 mM imidazole and 700 mM imidazole with 1% Triton X-100 respectively.

The purity of the recombinant proteins were monitored by SDS-PAGE, and protein concentrations were determined (DC Protein Assay, Bio-Rad, Hercules, CA, USA). The recombinant proteins were verified by Western blotting, using Anti Xpress antibody (Invitrogen) as primary antibody (1:5000) and Anti-mouse IgG-HRP (GE Healthcare, Little Chalfont, Buckinghamshire, UK) as secondary antibody (1:50 000) and detected by chemiluminescence (Amersham ECL Plus, GE Healthcare).

The purified recombinant proteins were used for immunization of one rabbit per protein; first injection using Freund's complete adjuvant and boosted three times with Freund's incomplete adjuvant weekly or with two weeks interval. The amount of μ1C- and σ1-antigen used per immunization was in the range of 70-920 μg and 110-240 μg respectively. The two antisera generated were named Anti-μ1c (rabbit # K265) and Anti-σ1 (rabbit # K275). The rabbit sera produced were tested by Western blotting using a purified batch of the respective recombinant protein as antigen (350 ng μ1C-protein and 189 ng σ1-protein). The generated antisera were used as primary antibody (1:1000), Anti-rabbit IgG-HRP (GE Healthcare) as secondary antibody (1:100 000) and the protein bands were detected by chemiluminescence (Amersham ECL Plus, GE Healthcare).

### Immunohistochemistry

Paraffin embedded sections on poly L-lysine coated glass slides were heated at 60 °C for 20 min, deparaffinized in xylene and rehydrated through graded alcohols. Antigen retrieval was performed by microwave treatment for 2X 6 min in citrate buffer (0.1 M, pH 6.0). Non-specific binding sites were blocked by goat serum diluted 1:50 in 5% skimmed dry milk in TBS [pH 7.6, 0.05 M Tris/HCl, 0.15 M NaCl] for 20 min. Primary antibody diluted in 1% skimmed milk in TBS was added and incubated in a humidity chamber at 4 °C overnight. Both the Anti-μ1C and Anti-σ1 sera were diluted 1:3000. The Anti-μ1C serum had been adsorbed by incubation on a monolayer of acetone-fixed Atlantic salmon head kidney (ASK) cell culture prior to use. A Vectastain ABC-AP kit (Vector Laboratories, Burlingame, CA, USA) was used for detection of bound antibody according to the manufacturer's instructions, employing Fast Red (1 mg mL^-1^) and Naphtol AS-MX phosphate (0.2 mg mL^-1^) with 1 mM Levamisole in 0.1 M TBS (pH 8.2) as substrate. Finally the sections were counterstained with Harris haematoxylin and mounted (Aquamount). All incubations, except with the primary antibodies, were carried out at room temperature in a humidity chamber.

Heart samples from Atlantic salmon collected during a field outbreak of HSMI were used as positive controls. The diagnosis had been set based upon histopathological criteria, and the samples had been confirmed as PRV positive by RT-qPCR. Samples from healthy Atlantic salmon, verified PRV negative by RT-qPCR, were used as negative controls. Primary and secondary antibody controls were performed by replacing the antibody with the respective diluent alone. Antisera controls were performed using rabbit serum collected prior to immunization and also by replacing the rabbit Anti-σ1 or Anti-μ1C serum with a rabbit serum raised against infectious pancreatic necrosis virus (IPNV). Absorption controls were performed by adding diluted immune serum (μ1C 1:3000, σ1 1:3000) to a solution of the corresponding recombinant protein serially diluted from 10 to 0.05 μg/mL in TBS with 1% BSA. The antigens were incubated with the immune sera for 12 h at 4 °C prior to use.

### Immunohistochemical scoring

The IHC staining was quantitatively assessed by one investigator in a blinded fashion. For each heart ventricle five non-overlapping fields of vision (FOV) were evaluated with a 20× objective using a light microscopy. Three of the FOVs were randomly selected from the outer part of the ventricle, comprising the epicardium, compact layer and various amounts of the spongy layer, and two FOVs were randomly selected from the spongy layer. The number of positive myocytes and leukocyte-like cells were counted separately in all five FOV. The amount of blood accumulated outside the ventricle varied between the slides, possibly affecting the number of positive leukocyte-like cells. Therefore, if any number of positive leukocyte-like cells were detected outside the ventricle in a FOV they were only counted as one positive cell. The number of positive cardiomyocytes and leukocyte-like cells counted in all five FOV were summarized separately and expressed as the arithmetic mean of positive myocytes and leukocyte-like cells per FOV (*n *= 5).

The FOV results for positive leukocyte-like cells and cardiomyocytes were subsequently used to assign two IHC-scores on a 6 point scoring scale from 0 to 5 based on the mean number of positive cells per FOV: 0 (none) 1 (0.1 to 1.0), 2 (1.1 to 5.0), 3 (5.1 to 20.0), 4 (20.1 to 50.0) and 5 (> 50.0). Finally, these individual IHC-scores were used to calculate the mean IHC-score ± standard deviation (SD) at each time of sampling (*n *= 5) for both the myocytes and leukocyte-like cell staining. The IHC scoring was performed for the Anti-σ1 and the Anti-μ1C stained sections.

Sequential tissue sections were used to evaluate the localization of the Anti-σ1 and Anti-μ1C staining with respect to each other. Morphological structures in the adjacent tissue sections were identified and used as a reference for orientation and evaluation of the staining.

### Histopathological scoring

Individual data was not available from the original study [2] which would have allowed us to directly link IHC scores to diseased or non-diseased fish. The IHC-stained sections were therefore re-examined for histopathological changes. The IHC sections were assessed in a blinded fashion by one investigator and scored using criteria described in Table 2 discriminating between epicardial and myocardial changes. The individual histopathological scores were used to calculate the mean score ± SD at each time of sampling (*n *= 5) for both epicardial and myocardial changes.

**Table 2:** Scoring description for histopathological examination of the sections.

Pathological description - epicard	Pathological description - myocard
**Score 0: **No pathological changes observed.	**Score 0: **No pathological changes observed.

**Score 0.1-0.9**: Focal/multifocal (2-4 foci) of inflammatory cells lifting the epicardial layer from the surface of the heart, typically 2-3 cell layers thick. Limited number (countable) of mononuclear inflammatory cells infiltrating the epicardium.	**Score 0.1-0.9**. Vascular changes in the small vessels of the compact layer characterized by enlarged endothelial cells, typically stretching out. Minor inflammatory changes of the compact layer without significant involvement of the spongious layer.
If there is only involvement of epicard with minor or very little compact layer involvement; max 1.5 score (diffuse and >5 cell layer thick for most of the inflamed area).	

**Score 1-1.9**: Diffuse infiltration of inflammatory cells (mononuclear) >5 cell layers thick in most of the epicard present. The infiltration of cells is multifocal to diffuse and can involve parts of or the entire epicardium available for assessment.	**Score 1-1.9: **Focal to multifocal inflammatory foci (2-5 foci) of the compact layer and/or in the spongious part (2-5 foci). Extension typically seen along small vessels and perivascular infiltration.

**Score 2.0-2.9**: Diffuse infiltration of inflammatory cells (mononuclear) >10 cell layers thick in most of the epicard present. Moderate pathological changes consisting of high number (uncountable) of inflammatory cells in the epicardium.	**Score 2.0-2.9: **The changes in the compact layer are multifocal or diffuse in areas and typically concentrate along small blood vessels. Combined with multifocal to diffuse changes in the spongious layer.

**Score 3**: Diffusely thickened (>15 cell layers) epicard in more than ¾ of the layer present. Severe pathological changes characterized by intense infiltration of inflammatory cells in the epicardium.	**Score 3: **Widespread to diffuse infiltration of inflammatory cells in the compact layer and involving the spongious layer in a multifocal pattern. Degeneration and or necrosis of muscle fibers may be/are seen. Atrium can also be involved with inflammatory changes

The sections were scored by histological examination evaluating epicardial and myocardial changes separately. The changes observed were indicated on a visual analog scale (0 to 3) based on the criteria described in the Table.

## Results

### Protein expression and immunization

**Figure 1:**
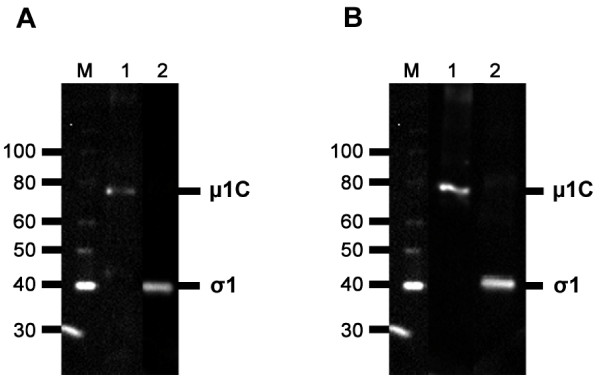
**Purified recombinant protein and specificity of rabbit antisera**. (A) Purified recombinant protein detected by Western blot using anti-Xpress epitope mAb as primary antibody. M, marker protein; Lane 1, recombinant protein μ1C detected at approximately 78 kDa; Lane 2, recombinant protein σ1 detected at approximately 40 kDa. (B) Western blot analysis of Anti-μ1C and Anti-σ1 rabbit sera detecting their respective recombinant proteins. The results corresponded to the bands detected in Figure 1a. M, marker protein; Lane 1, Anti-μ1C rabbit serum; Lane2, Anti-σ1 rabbit serum.

The μ1C and σ1 proteins were expressed in *E.coli *and visualized by SDS-PAGE at the expected sizes of 78 kD and 40 kD (data not shown). The presence of the His-tagged fusion proteins was verified by Western blotting using Anti-Xpress mAb recognizing the fusion peptide (Figure 1a). Western blotting also confirmed that the rabbit μ1C and σ1 antisera recognized their corresponding recombinant protein (Figure 1b). No staining was detected using the pre-immunization sera.

### Immunohistochemistry

The IHC analysis demonstrated the presence of PRV antigen in cardiomyocytes and leukocyte-like cells in heart tissue of Atlantic salmon. The specific IHC-staining in these cells was characterized by a distinct red even coloring of the cytoplasm. Stained cardiomyocytes were found both in the compact and spongy layer of the ventricle, while positive stained leukocyte-like cells were located in areas of clotted blood and in blood trapped in vessels or the heart lumen. A faint cytoplasmatic staining was occasionally detected in some red blood cells (data not shown). The mean scores (± SD) for IHC staining of cardiomyocytes and leukocyte-like cells and the histopathological changes observed at each time of sampling (*n *= 5) for the inoculated and cohabitant group are presented in Table 3. The individual data for each sample in the inoculated and cohabitant group are listed in Additional file 1: (Table S1).

**Table 3:** IHC- and histopathology mean scores.

A. Inoculated group						
	**IHC Anti-σ1**		**IHC Anti-μ1C**		**Histopathology**	
	
**Wpi**	**Leuk**.	**Myocyte**	**Leuk**.	**Myocyte**	**Epicard**	**Myocard**

2 wpi	0 ± 0	0 ± 0	0 ± 0	0 ± 0	0.20 ± 0.12	0 ± 0
4 wpi	1.00 ± 0.71	0 ± 0	0.60 ± 0.55	0 ± 0	0.14 ± 0.19	0 ± 0
6 wpi	1.40 ± 0.89	1.40 ± 1.14	0.60 ± 0.55	1.40 ± 1.52	1.18 ± 0.56	0.16 ± 0.26
8 wpi	0.60 ± 0.55	1.60 ± 0.55	0.20 ± 0.45	1.40 ± 0.55	1.68 ± 0.45	0.80 ± 0.49
10 wpi	0 ± 0	0.40 ± 0.55	0 ± 0	0.60 ± 0.55	1.60 ± 0.57	0.60 ± 0.55
12 wpi	0 ± 0	0.20 ± 0.45	0 ± 0	0.20 ± 0.45	0.62 ± 0.29	0.94 ± 0.17
**B. Cohabitant group**						

	**IHC Anti-σ1**		**IHC Anti-μ1C**		**Histopathology**	
	
**Wpi**	Leuk.	Myocyte	Leuk.	Myocyte	Epicard	Myocard

6 wpi	0 ± 0	0 ± 0	0 ± 0	0 ± 0	0.34 ± 0.16	0.06 ± 0.06
8 wpi	1.80 ± 1.10	0 ± 0	1.40 ± 0.89	0 ± 0	0.58 ± 0.37	0 ± 0
10 wpi	1.40 ± 0.89	3.20 ± 1.64	0.80 ± 0.83	3.20 ± 1.30	1.90 ± 0.60	0.56 ± 0.46
12 wpi	0 ± 0	1.80 ± 0.84	0 ± 0	2.00 ± 1.00	1.94 ± 0.54	1.7 ± 0.43

Data represent mean scores (± SD) for IHC staining and histopathological changes at each time of sampling (*n *= 5) shown for the inoculated (A) and cohabitant group (B). The IHC staining was performed with both Anti-σ1 and Anti-μ1C and scored (0-5) for positive leukocyte-like cells and cardiomyocytes. The histological changes were scored (0-3) for epicardial- and myocardial changes.

### IHC controls

Staining was not observed in any samples of fish from the negative control group of the experimental trial. No staining was detected in the antibody- (primary and secondary) or antiserum controls. Absorption of the two sera with their respective purified recombinant protein blocked the immunostaining. The staining gradually reappeared as the concentration of the antigen used for absorption was reduced (data not shown).

### Comparing Anti-σ1 and Anti-μ1C

The staining patterns were found to be similar for the σ1 and the μ1C antisera, including no major differences in the distribution or in number of stained cells. Positive staining was detected in the same histological regions using the two sera on sequential sections (data not shown). The only notable tendency was that that the staining with σ1 antiserum had slightly higher mean score for leukocyte-like cells compared to sections stained with μ1C antiserum (Table 3). However, this did not affect the overall trend observed in the study. The following mean scores for IHC-staining referred in the text will correspond to the values obtained for the σ1 antiserum staining, unless otherwise stated.

### Inoculated group

**Figure 2:**
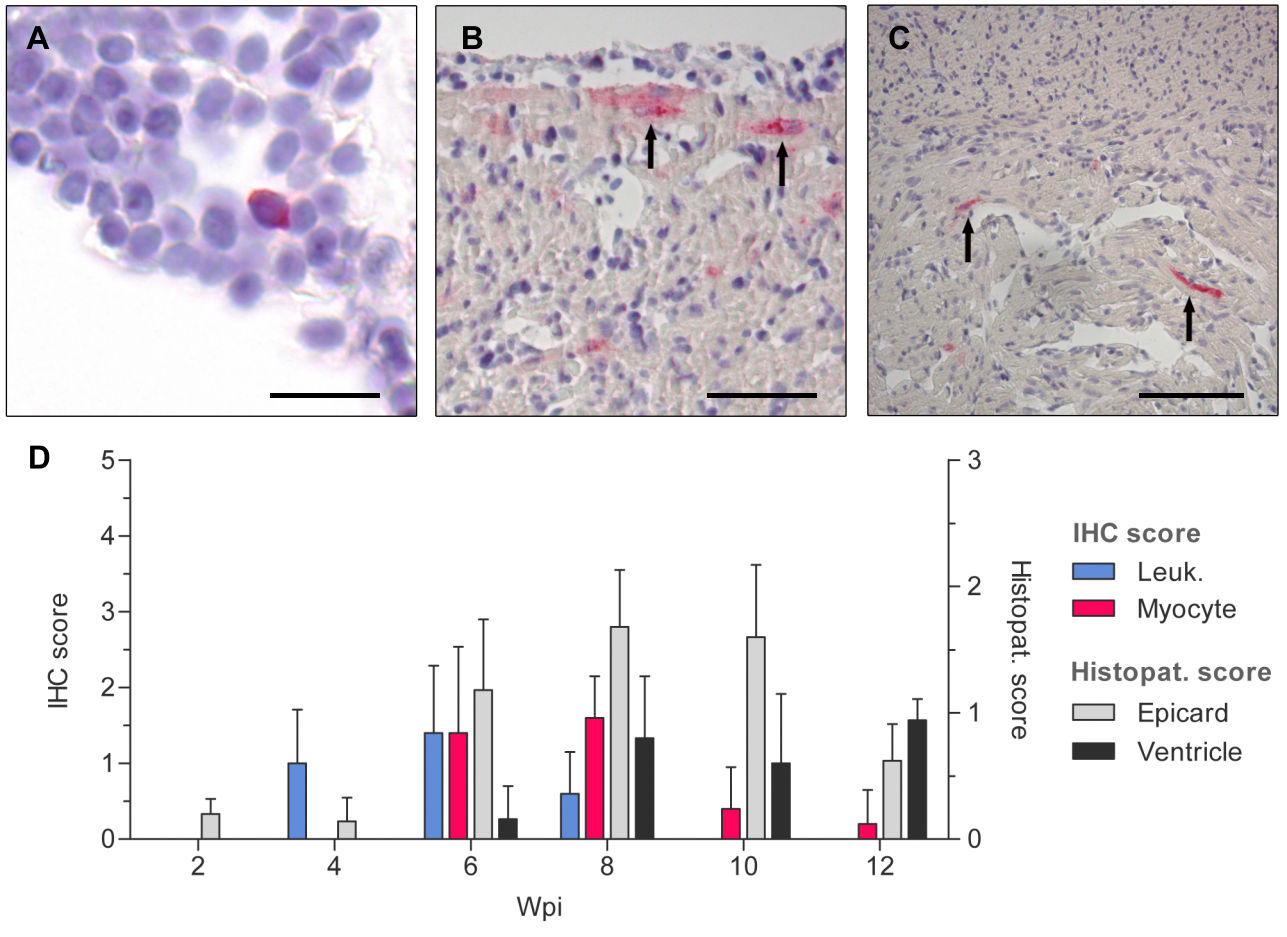
**IHC and histopathology for the inoculated group**. Immunostaining of heart tissue sections and graphical presentation of the IHC- and histopathological scores from the inoculated group. (**A**) Blood clot in heart 4 wpi: PRV antigen detected with σ1-antibodies in a single leukocyte-like cell observed as red cytoplasmatic staining. Error bar 15 μm. (**B**) Heart ventricle 6 wpi: Immunostained cardiomyocytes detected with μ1c-antibodies in the outer part of the compact layer (arrow). Error bar 50 μm. (**C**) Heart ventricle 8 wpi: Scattered immunostained cardiomyocytes (arrow) observed in the inner part of compact layer and in the spongy layer. No positive cells detected within the inflamed outer compactum in the top half of the picture. Section stained with σ1-antibodies. Error bar 100 μm. (**D**) The graph illustrates the IHC-staining with σ1 antiserum and the histopathological changes at each time of sampling (Wpi). IHC-scores (left Y-axis) are shown for leukocyte-like cells (blue) and cardiomyocytes (red). Histopathological score (right Y-axis) are presented for epicardial-(gray) and ventricular changes (black). All bars represent mean values (+SD) for each group (*n *= 5).

Antigen specific for PRV was first detected by IHC in leukocyte-like cells of the heart at 4 wpi. At this time point immunostaining was observed within a few scattered leukocyte-like cells in four of the five sampled fish with a mean score of 1.00 ± 0.71 (Figure 2a). At 6 wpi four sampled fish were positive for PRV antigen in leukocyte-like cells with an increased mean score (1.40 ± 0.89). Up until this point only minor histopathological changes had been observed, but at 6 wpi all the samples were scored with marked epicardial changes (1.18 ± 0.56). All scoring results are presented graphically in Figure 2d. The positive leukocyte like cells were very rarely located within the inflamed epicardium, but appeared more frequently in vessels or the ventricular lumen. Some positive cells were detected adjacent to the endothelium lining the luminal side of the spongy layer. In addition, what appeared to be stained endothelial cells were occasionally detected in slides with high leukocyte-like cell score. At 6 wpi viral antigen was also detected in cardiomyocytes for the first time, and it was observed in four of the five fish examined (1.40 ± 1.14). The immunostaining of cardiomyocytes were located to the compact layer as well as in the spongy layer of the ventricle (Figure 2b).

At 8 wpi PRV antigen in cardiomyocytes were observed in all of the five fish sampled and peaked with a slightly increased mean score (1.60 ± 0.55). In contrast, the amount of PRV antigen in leukocyte-like cells was reduced and only detected in a few cells in three fish (mean score 0.6 ± 0.55). An inflammatory response was evident in the outer part of the compact layer at this time point. The histopathology scoring showed an increased epicardial score (1.68 ± 0.45) and myocardial changes were now also present in all samples (mean score 0.8 ± 0.55). The PRV stained cardiomyocytes were found away from the inflamed outer compactum in the unaffected inner compact layer and also in the spongy layer (Figure 2c). In general, little or no cellular changes were observed in the positive stained cardiomyocytes.

At 10 wpi two of the five sampled fish stained in the cardiomyocytes but with reduced mean score (0.4 ± 0.55). No staining in the leukocyte-like cells could be detected at this time point. Histopathological changes were still evident in the epicardium as well as the myocardium. Similar to the observation at 8 wpi, the immunostained cardiomyocytes at 10 wpi were absent in the most inflamed outer compact region, and positive stained cardiomyocytes were only observed in the inner compact and the spongy layer. At 12 wpi PRV antigen was detected in one out of five fish in which only one single myocyte was observed in all five fields of vision examined. The epicardial changes were clearly reduced (0.62 ± 0.29), but the myocardial changes were about the same level (0.94 ± 0.17) (Figure 2d).

### Cohabitant group

**Figure 3:**
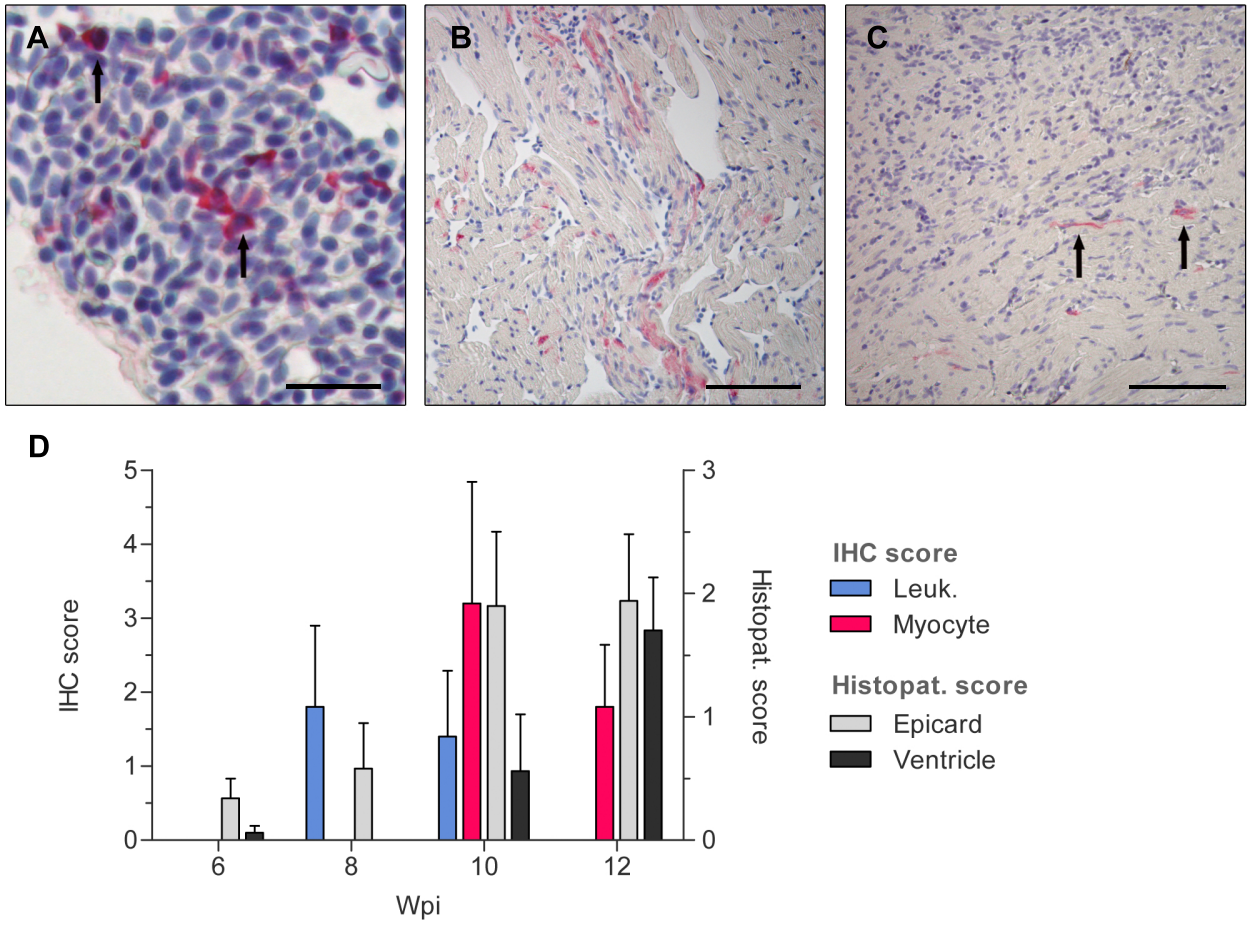
**IHC and histopathology for the cohabitant group**. Immunostaining of heart tissue sections and graphical presentation of the IHC- and histopathological scores from the cohabitant group. (**A**) Blood clot in heart 8 wpi: PRV antigen detected with σ1-antibodies in numerous leukocyte-like cell (arrow). Error bar 25 μm. (**B**) Heart ventricle 10 wpi: A high number of immunostained cardiomyocytes in the spongy layer. Section stained with σ1-antibodies. Error bar 100 μm. (**C**) Heart ventricle 12 wpi: Scattered immunostained cardiomyocytes (arrow) detected with σ1-antibodies in the inner part of compact layer. Inflammation response in the outer part of the compact layer seen in the top half of the picture. Error bar 100 μm. (**D**) The graph illustrates the IHC staining with Anti-σ1 and the histopathological changes at each time of sampling (Wpi). IHC staining scores (left Y-axis) are indicated for leukocyte-like cells (blue) and cardiomyocytes (red). Histopathological scores (right Y-axis) are presented for epicardial- (gray) and ventricular changes (black). All bars represent mean values (+SD) for each group (*n *= 5).

The staining pattern in the cohabitant group resembled that of the inoculated group, but with a delayed onset of 2-4 weeks. The first PRV antigen was detected 8 wpi when four of five fish stained positive in leukocyte-like cells with a high mean score (1.80 ± 1.10). As for the inoculated group, some positive leukocyte-like cells appeared adjacent to the endothelium in the ventricular lumen and some cells looking like endothelial cells stained positive for PRV. Minor epicardial changes were now detected (0.58 ± 0.37), but none of the samples scored above 1.0. All scoring results are presented graphically in Figure 3d.

**Figure 4:**
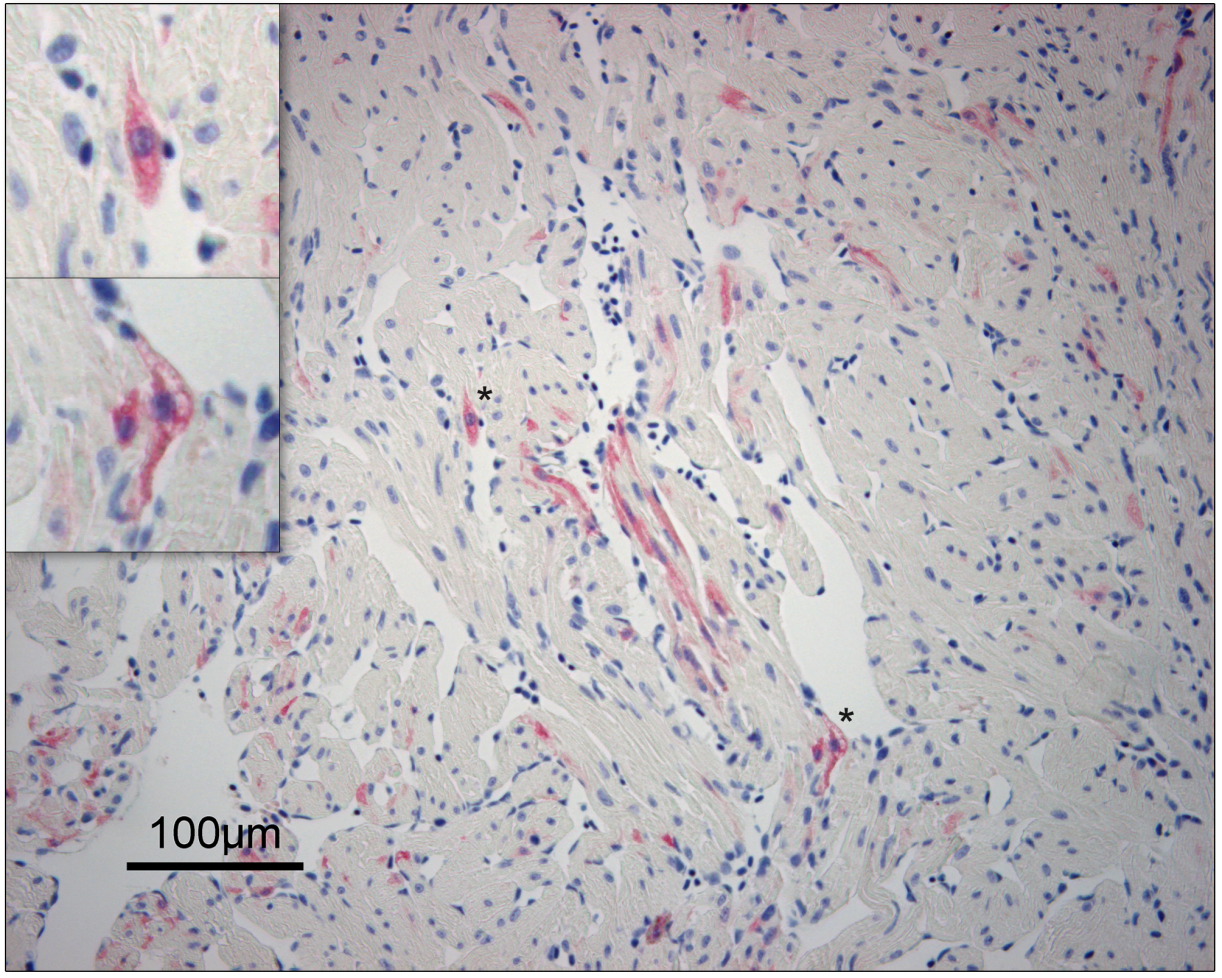
**Peak staining of cardiomyocytes**. Immunostaining with σ1-antibodies of heart section from the cohabitant group 10 wpi. PRV antigen was detected in numerous cardiomyocytes in the ventricle (red color). This represents the time of peak staining in the cohabitant group and positive staining was observed in both the compact (right side) and spongy layer (mid to left side). Magnified sections (*) in the top left corner clearly show the cytoplasmatic staining of the cardiomyocytes outlining the nucleus. Vacuolization in a positive stained cardiomyocyte is visible in the bottom magnified picture.

At 10 wpi positive cardiomyocytes were detected in all fish sampled with a high mean score (3.20 ± 1.64) (Figure 4). These positively stained cells were found throughout the compact as well as the spongy layer in all five fish examined (Figure 3b). This marked infection of cardiomyocytes coincided with highly elevated epicardial changes with a mean histopathological score of 1.90 ± 0.60. Myocardial changes were now also detected, though at a relatively low level (0.56 ± 0.46). As in the inoculated group, the cardiomyocytes positive for PRV were observed in cells with little or no cellular changes. The mean score for leukocyte-like cells were starting to decrease at 10 wpi (1.40 ± 0.89) compared to 8 wpi.

**Figure 5:**
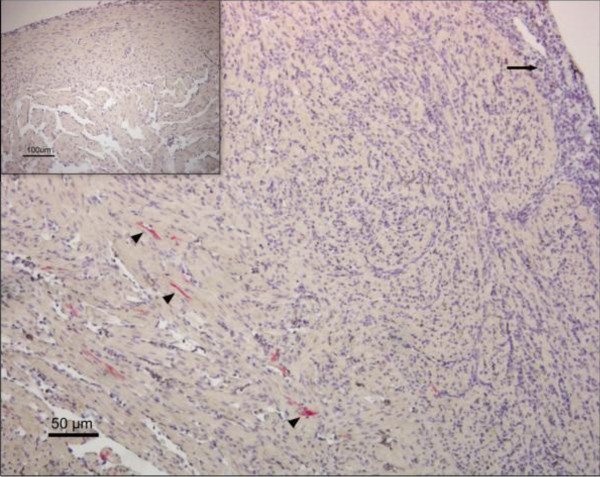
**IHC staining and classical HSMI changes**. Immunostaining with μ1c-antibodies of heart section from the cohabitant group 12 wpi. Characteristic histopathological changes of HSMI with epicarditis (arrow) and a major inflammatory response in the outer part of the compact layer. No positive immunostained cells observed within the inflammatory reaction. PRV antigen is detected outside the area of inflammation (arrowhead) in the inner part of the compactum, as well as in the spongy layer (lower left corner). Negative control presented in the top left corner.

At 12 wpi none of the observed fish stained in the leukocytes. Positive cardiomyocytes were detected in all samples, but the mean score was now decreasing (1.80 ± 0.84) (Figure 3c). There was a substantial inflammatory response in the compact layer with a high epicardial mean score (1.94 ± 0.54), and the myocardial changes was now also highly prominent (1.7 ± 0.43). The cardiomyocytes positive for PRV were located in the inner compact layer away from the area of inflammation and also in the spongy layer (Figure 5).

The atrium and bulbus arteriosus of the heart was not present in all the samples and therefore not included in this study. However, in some of the samples where the atrium was present positive cardiomyocyte staining was detected. This was observed in samples with high ventricle scores. No staining was detected in the bulbus arteriosus.

## Discussion

In this study we used IHC to demonstrate the presence of PRV antigens in heart tissue of Atlantic salmon with HSMI. The PRV antigens were detected in the cytoplasm of leukocyte-like cells and cardiomyocytes, which is in agreement with the cytoplasmic replication of reoviruses. The positive staining first appeared in leukocyte-like cells in the heart, and later in cardiomyocytes. A similar sequential staining pattern was observed in both the inoculated-and cohabitant group, with a 4 weeks delay in onset of detection in the cohabitant group. In both groups an initial rise and then a subsequent fall in viral load were observed.

The first detection of PRV antigen by IHC predated the initial histopathological changes by two weeks as PRV antigen was detected in leukocyte-like cells at 4 wpi and 8 wpi in the two respective groups. Two weeks later (at 6 wpi and 10 wpi) PRV antigen was also observed in cardiomyocytes coinciding with the occurrence of significant epicardial changes, likely triggered by viral replication (Figure 2d, Figure 3d). Our IHC and histopathology results correlated with the previous histopathological study of the same material, where the first lesions were reported at 6 and 10 wpi in the inoculated and cohabitant group, respectively [2]. In the former study the sampled fish were classified as diseased or non-diseased based on histopathological lesions of the heart consistent with HSMI. In our study the sampled fish were scored from 0-3 on a continuous scale for both epicardial and myocardial changes allowing us to monitor both the location and development of histopathological lesions during the disease and linking these findings to the IHC-staining.

In the inoculated group the positive staining for PRV of leukocyte-like cells culminated approximately 6 wpi, while a similar peak of cardiomyocytes was delayed by 2 weeks. The same pattern was observed in the cohabitant group but with sharper increase and higher mean score for both leukocyte-like cells and cardiomyocytes (Figure 3d). The sharper increase and higher mean score for IHC staining correlated with the recorded epicardial and myocardial changes in the cohabitant group, linking the number of infected cells with severity of histopathological lesions. The abrupt increase might be attributed to the route of infection (cohabitant challenge) which would mimic a natural and probably more efficient infection.

In the present study PRV-antigen was detected in leukocyte-like cells. For avian orthoreoviruses (ARV), macrophages have been proposed as a target cell type for viral replication [19-21], and there is evidence of ARV strain differences in the ability to replicate in cultured chicken leukocytes [20,22]. In our study it was a long period, i.e. 4-6 weeks, from inoculation to the appearance of PRV-antigen in cardiomyocytes and histopathological lesions in the heart. This delay could be due to a primary viral replication prior to these events, and the observed staining of leukocyte-like cells indicates that these might be early target cells for PRV. Whether the detection of PRV antigen in leukocyte-like cells represents viral replication, passive transport or phagocytosis is unclear. Hematopoietic tissue in Atlantic salmon is primarily located to the head kidney and spleen. These organs should be investigated in future studies to further elucidate the viral spread in the initial stages of PRV infection.

One characteristic feature of HSMI is epicarditis. In the present study some positive leukocyte-like cells were observed in epicardial coronary arteries or among the inflammatory cells lifting the epicardial layer during epicarditis. However, no positive immunohistochemical staining of the epicardial epithelium was detected in this study. Generally, most of the PRV positive leukocyte-like cells were observed in clots of blood, vessels or the ventricular lumen. The ventricular lumen is lined with endothelium, and some of the positive cells appeared adjacent to or attached to the endothelium. In addition, a few cells resembling endothelial cells stained positive for PRV antigen. For mammalian orthoreoviruses (MRV), the viral attachment protein, σ1, binds to junctional adhesion molecule-A (JAM-A) and permits reovirus infection [23]. The JAM-A is a member of the immunoglobulin superfamily and is located at tight junctions in the epithelium and endothelium in mammals and also found on leukocytes and platelets [24]. It is demonstrated that JAM-A is required for hematogenous dissemination of MRV [25]. During an inflammatory response, JAM-A redistributes from cellular junctions to the apical surface and engages in transendothelial migration of circulating leukocytes [26,27]. Little is known about tight junction proteins in salmon, and no analog to JAM-A has been described. Although our findings indicate the sequence of the infection of leukocyte-like cells and cardiomyocytes, the transmission mechanism used by PRV between these cells requires further studies.

The number of leukocyte-like cells that stained positive for PRV decreased from 6 wpi and onwards, and no staining was observed in these cells at 10 wpi in the inoculated group. A similar decline in staining frequency was also observed in the cardiomyocytes. Generally, the stained cardiomyocytes were observed in cells with no apparent signs of cellular damage that where located to areas with little inflammation. However, there was a tendency that areas observed with cardiomyocyte staining were followed by histopathological changes at the next time of sampling. Up to 8 wpi in the inoculated group and 10 wpi in the cohabitant group positively stained cardiomyocytes were detected in the outer most regions of the compactum (Figure 2b) as well as the rest of the compact and spongy layer. As the infection and inflammatory response progressed, positively stained cardiomyocytes were only observed in the non-inflamed areas away from the inflammation in the compactum layer (Figure 2c, Figure 3c, Figure 5). This is in agreement with observations in field outbreaks of HSMI, where the most severe lesions shift from the compactum in early stages to the spongy layer at later stages [4].

There was a marked decline in the staining for PRV towards the end of the experiment. By histopathological examination it was observed that following the peak severity of epicardial lesions at 8 to 10 wpi, the changes at 12 wpi were more moderate in character. This is in agreement with the previous histopathological study of the same material [2]. Thus, there is a similar trend seen by IHC and with histopathological examination, indicative of the infection subsiding towards the end of the experimental period. Together, these results indicate that the cardiomyocyte infection under experimental conditions is transient and that the immune response reduces the viral load in the heart to a level below detection by immunohistochemistry. In this study it seems that the inflammatory response will result in the viral infection being fought off and further the observation from the fields that HSMI is appearing only once in a population of farmed salmon, could raise some promises as regards possibility for future development of prophylactic interventions.

Myocarditis associated with a reoviral infection has also been described in other intensive animal production systems, i.e. in turkey poults and chickens [12,14]. In turkey poults, reovirus antigen, detected by IHC, was found in both cardiomyocytes and mononuclear inflammatory cells of the heart. Microscopically, they found mild to severe necrosis of myocytes and infiltration of primarily lymphocytes [14]. MRV induced myocarditis in mice has been a widely used model for the study of viral myocarditis. MRV strains differ in their myocarditic potential, and multiple viral core proteins have been suggested to be determinants of myocarditis [16-18,28-30]. It is not known if there are variation in pathogenicity or myocarditic potential between strains of PRV. This would have to be a subject for future study.

The immunohistochemical detection of PRV is an informative, supportive tool for diagnostic histopathology of HSMI. Currently histopathological findings are used for the diagnosis of HSMI, but immunohistochemistry would be beneficial as a supplementary diagnostic tool. At the end of the experiment myocardial changes were still present in the inoculated group but with little or no PRV detected by IHC. This could be an important point when interpreting IHC staining, especially if the samples originate from field outbreaks. A longitudinal study of a natural outbreak of HSMI showed longer duration of the disease [4], and samples collected from natural outbreaks would likely be at different stages of the disease. Among these, one would expect to find HSMI diseased fish with characteristic lesion in the absence of positive IHC, representing later stages of the disease. This highlights the importance for early sampling in order to detect PRV by immunohistochemistry during the onset of disease.

PRV is almost ubiquitously present in Atlantic salmon marine farms [9], and detection of PRV alone does not establish an HSMI diagnosis. The PRV load is, however, correlated with HSMI [9], and in field outbreaks of HSMI the histopathological changes are more severe than those observed in experimental infections. This indicates that non-PRV factors such as stress and other concurrent diseases may contribute to development of HSMI. Atlantic salmon aquaculture confines age-uniform single species populations at high stocking density, conditions that favor transmission of infectious agents and reduce resistance to disease due to stress. The ubiquity of PRV and increasing number of HSMI outbreaks may indicate selective pressure for efficiently spreading and possibly more virulent virus strains.

In conclusion, the viral distribution in heart tissue during an experimental infection of HSMI in Atlantic salmon showed a sequential staining pattern of PRV antigen in leukocyte-like cells and in ventricular cardiomyocytes. Our results confirm the association between PRV and HSMI, and strengthen the hypothesis of PRV being the causative agent of HSMI. Immunohistochemical detection of PRV antigens can be beneficial for the understanding of the pathogenesis of HSMI as well as for diagnostic purposes.

## Abbreviations

FOV: Fields of vision; IHC: Immunohistochemistry; JAM-A: Junctional adhesion molecule-A; HSMI: Heart and skeletal muscle inflammation; MRV: Mammalian orthoreovirus; PRV: Piscine reovirus.

## Competing interests

The authors declare that they have no competing interests.

## Authors' contributions

ER participated in the overall design and coordination of the study and helped in interpretation of data and drafting the manuscript. KF participated in the design and interpretation of the immunohistochemistry and revised the manuscript. ML designed and performed the cloning, and revised the manuscript. ØE designed and carried out the histopathological scoring, and revised the manuscript. ØWF participated in the overall design of the study, performed and interpreted expression, immunization and immunohistochemical staining and drafted the manuscript. All authors read and approved the final manuscript.

## Supplementary Material

Additional file 1 **Table S1 **IHC- and histopathology data. Shown are individual data for IHC staining and histopathological changes at each time of sampling for the inoculated group (A) and the cohabitant group (B). The IHC staining was performed with both Anti-σ1 and Anti-μ1C and positive leukocyte-like cell and cardiomyocytes per field of vision (FOV) was counted. These results were subsequently used to assign an IHC-score (0-5) based of the following categorization of positive cells per FOV: 0 (none) 1 (0.1 to 1.0), 2 (1.1 to 5.0), 3 (5.1 to 20.0), 4 (20.1 to 50.0) and 5 (> 50.0). The histological changes were scored (0-3) for both epicardial- and myocardial changes. Click here for file
