# Supplementary material for: Immunohistochemical detection of piscine reovirus (PRV) in hearts of Atlantic salmon coincide with the course of heart and skeletal muscle inflammation (HSMI)
Source: Vet Res. 2012 Apr 9;43(1):27. doi: 10.1186/1297-9716-43-27 (PMC3384478; doi:10.1186/1297-9716-43-27)
Supplement: Additional file 1 — Table S1 IHC- and histopathology data. Shown are individual data for IHC staining and histopathological changes at each time of sampling for the inoculated group (A) and the cohabitant group (B). The IHC staining was performed with both Anti-σ1 and Anti-μ1C and positive leukocyte-like cell and cardiomyocytes per field of vision (FOV) was counted. These results were subsequently used to assign an IHC-score (0-5) based of the following categorization of positive cells per FOV: 0 (none) 1 (0.1 to 1.0), 2 (1.1 to 5.0), 3 (5.1 to 20.0), 4 (20.1 to 50.0) and 5 (> 50.0). The histological changes were scored (0-3) for both epicardial- and myocardial changes. [file 1297-9716-43-27-S1.DOC]

1. **Inoculated group**

|  |  | **IHC Anti-σ1** | | | |  | **IHC Anti-μ1C** | | | |  | **Histopathology** | |
| --- | --- | --- | --- | --- | --- | --- | --- | --- | --- | --- | --- | --- | --- |
| **Wpi** | **Fish** | **Leuk.** Score (FOV) | | **Myocyte** Score (FOV) | |  | **Leuk.** Score (FOV) | | **Myocyte** Score (FOV) | |  | **Epicard** Score | **Myocard** Score |
|  |  |  |  |  |  |  |  |  |  |  |  |  |  |
| 2  wpi | L1 | 0 | (0) | 0 | (0) |  | 0 | (0) | 0 | (0) |  | 0.3 | 0 |
|  | L2 | 0 | (0) | 0 | (0) |  | 0 | (0) | 0 | (0) |  | 0.2 | 0 |
|  | L3 | 0 | (0) | 0 | (0) |  | 0 | (0) | 0 | (0) |  | 0.2 | 0 |
|  | L4 | 0 | (0) | 0 | (0) |  | 0 | (0) | 0 | (0) |  | 0 | 0 |
|  | L5 | 0 | (0) | 0 | (0) |  | 0 | (0) | 0 | (0) |  | 0.3 | 0 |
| 4  wpi | L1 | 0 | (0) | 0 | (0) |  | 0 | (0) | 0 | (0) |  | 0.4 | 0 |
|  | L2 | 1 | (1.0) | 0 | (0) |  | 1 | (0.2) | 0 | (0) |  | 0.3 | 0 |
|  | L3 | 1 | (0.2) | 0 | (0) |  | 0 | (0) | 0 | (0) |  | 0 | 0 |
|  | L4 | 2 | (3.6) | 0 | (0) |  | 1 | (0.8) | 0 | (0) |  | 0 | 0 |
|  | L5 | 1 | (0.2) | 0 | (0) |  | 1 | (0.2) | 0 | (0) |  | 0 | 0 |
| 6  wpi | L1 | 1 | (0.8) | 1 | (0.2) |  | 0 | (0) | 1 | (0.2) |  | 1 | 0 |
|  | L2 | 2 | (3.2) | 0 | (0) |  | 1 | (0.6) | 0 | (0) |  | 0.5 | 0 |
|  | L3 | 2 | (1.4) | 3 | (19.0) |  | 1 | (0.6) | 3 | (21.4) |  | 1.9 | 0.6 |
|  | L4 | 2 | (3.2) | 1 | (0.2) |  | 1 | (0.6) | 1 | (0.4) |  | 1.6 | 0.2 |
|  | L5 | 0 | (0) | 2 | (1.2) |  | 0 | (0) | 1 | (0.8) |  | 0.9 | 0 |
| 8  wpi | L1 | 1 | (0.4) | 1 | (0.2) |  | 1 | (0.2) | 1 | (0.2) |  | 1.1 | 0.1 |
|  | L2 | 0 | (0) | 1 | (0.4) |  | 0 | (0) | 1 | (0.8) |  | 1.3 | 0.8 |
|  | L3 | 1 | (0.2) | 2 | (1.8) |  | 0 | (0) | 2 | (2.2) |  | 1.9 | 0.6 |
|  | L4 | 0 | (0) | 2 | (1.8) |  | 0 | (0) | 2 | (1.4) |  | 2.1 | 1.1 |
|  | L5 | 1 | (0.6) | 2 | (1.6) |  | 0 | (0) | 1 | (0.6) |  | 2 | 1.4 |
| 10 wpi | L1 | 0 | (0) | 0 | (0) |  | 0 | (0) | 1 | (0.2) |  | 1 | 0 |
|  | L2 | 0 | (0) | 1 | (0.2) |  | 0 | (0) | 1 | (0.8) |  | 2 | 0.6 |
|  | L3 | 0 | (0) | 1 | (0.2) |  | 0 | (0) | 0 | (0) |  | 1 | 0.1 |
|  | L4 | 0 | (0) | 0 | (0) |  | 0 | (0) | 0 | (0) |  | 2.2 | 1.1 |
|  | L5 | 0 | (0) | 0 | (0) |  | 0 | (0) | 1 | (0.2) |  | 1.8 | 1.2 |
| 12 wpi | L1 | 0 | (0) | 0 | (0) |  | 0 | (0) | 0 | (0) |  | 1 | 0.9 |
|  | L2 | 0 | (0) | 0 | (0) |  | 0 | (0) | 0 | (0) |  | 0.6 | 1 |
|  | L3 | 0 | (0) | 1 | (0.2) |  | 0 | (0) | 1 | (0.2) |  | 0.6 | 1.2 |
|  | L4 | 0 | (0) | 0 | (0) |  | 0 | (0) | 0 | (0) |  | 0.7 | 0.8 |
|  | L5 | 0 | (0) | 0 | (0) |  | 0 | (0) | 0 | (0) |  | 0.2 | 0.8 |

B. Cohabitant group

|  |  | **IHC Anti σ1** | | | |  | **IHC Anti μ1C** | | | |  | **Histopathology** | |
| --- | --- | --- | --- | --- | --- | --- | --- | --- | --- | --- | --- | --- | --- |
| **Wpi** | **Fish** | **Leuk.** Score (FOV) | | **Myocyte** Score (FOV) | |  | **Leuk.** Score (FOV) | | **Myocyte** Score (FOV) | |  | **Epicard** Score | **Myocard** Score |
|  |  |  |  |  |  |  |  |  |  |  |  |  |  |
| 6  wpi | L1 | 0 | (0) | 0 | (0) |  | 0 | (0) | 0 | (0) |  | 0.3 | 0 |
|  | L2 | 0 | (0) | 0 | (0) |  | 0 | (0) | 0 | (0) |  | 0.4 | 0.1 |
|  | L3 | 0 | (0) | 0 | (0) |  | 0 | (0) | 0 | (0) |  | 0.6 | 0.1 |
|  | L4 | 0 | (0) | 0 | (0) |  | 0 | (0) | 0 | (0) |  | 0.2 | 0 |
|  | L5 | 0 | (0) | 0 | (0) |  | 0 | (0) | 0 | (0) |  | 0.2 | 0.1 |
| 8  wpi | L1 | 3 | (5.8) | 0 | (0) |  | 2 | (4.6) | 0 | (0) |  | 0.8 | 0 |
|  | L2 | 2 | (4.4) | 0 | (0) |  | 1 | (0.8) | 0 | (0) |  | 0.6 | 0 |
|  | L3 | 0 | (0) | 0 | (0) |  | 0 | (0) | 0 | (0) |  | 0 | 0 |
|  | L4 | 2 | (3.4) | 0 | (0) |  | 2 | (3.8) | 0 | (0) |  | 0.5 | 0 |
|  | L5 | 2 | (2.8) | 0 | (0) |  | 2 | (1.4) | 0 | (0) |  | 1 | 0 |
| 10 wpi | L1 | 1 | (0.2) | 1 | (0.6) |  | 0 | (0) | 2 | (1.8) |  | 2.1 | 1 |
|  | L2 | 2 | (1.6) | 4 | (37.4) |  | 2 | (1.2) | 4 | (43.0) |  | 1 | 0.2 |
|  | L3 | 2 | (2.6) | 4 | (22.4) |  | 1 | (0.4) | 3 | (18.4) |  | 2.3 | 1 |
|  | L4 | 0 | (0) | 2 | (1.8) |  | 0 | (0) | 2 | (1.8) |  | 2.5 | 0.6 |
|  | L5 | 2 | (1.2) | 5 | (74.4) |  | 1 | (0.4) | 5 | (69.8) |  | 1.6 | 0 |
| 12 wpi | L1 | 0 | (0) | 1 | (0.4) |  | 0 | (0) | 1 | (0.2) |  | 2.1 | 2 |
|  | L2 | 0 | (0) | 3 | (11.6) |  | 0 | (0) | 3 | (19.0) |  | 2.3 | 2.1 |
|  | L3 | 0 | (0) | 1 | (0.4) |  | 0 | (0) | 1 | (0.4) |  | 2.3 | 1.1 |
|  | L4 | 0 | (0) | 2 | (3.2) |  | 0 | (0) | 3 | (6.0) |  | 2 | 1.9 |
|  | L5 | 0 | (0) | 2 | (1.6) |  | 0 | (0) | 2 | (2.2) |  | 1 | 1.4 |
